# Outcome, Pain Perception, and Health-Related Quality of Life in Patients Submitted to Percutaneous Ethanol Injection for Simple Thyroid Cysts

**DOI:** 10.1155/2017/9536479

**Published:** 2017-07-09

**Authors:** Roberto Negro, Ermenegildo Colosimo, Gabriele Greco

**Affiliations:** ^1^Division of Endocrinology, “V. Fazzi” Hospital, Piazza F. Muratore, 73100 Lecce, Italy; ^2^Division of Pathology, “V. Fazzi” Hospital, Piazza F. Muratore, 73100 Lecce, Italy

## Abstract

Thyroid cysts are usually benign lesions that when voluminous may induce cosmetic concerns or local discomfort. Percutaneous ethanol injection (PEI) has been demonstrated to be effective for shrinkage of such cysts. In this retrospective study, we evaluated the efficacy, pain perception, and health-related quality of life (HRQL) in patients submitted to PEI for pure cystic lesions. We reviewed the data of 101 patients who underwent ≤3 PEI. In the whole group of patients, the volume reduction was 66% after the first, 74.4% after the second, and 79.4% after the third PEI treatment. 55.4% had a cystic volume ≤ 10 ml; 85.7% of cysts ≤ 10 ml were cured by just one PEI. The number of PEI was significantly higher in the >30.0 ml group; this latter group obtained the smallest percent reduction versus baseline after the first PEI when compared with smaller cysts. The sensation of pain reported during PEI was absent in 78.3% of cases, and HRQL significantly improved from pre- to the posttreatment. PEI is a safe and effective technique for pure cystic lesions. In addition, HRQL significantly improves, providing a further support for this procedure.

## 1. Introduction

Thyroid cysts, that is, a thyroid lesion having a delimiting wall with a fluid content, are more rare than thyroid nodules but may induce cosmetic concerns and compressive symptoms as well. They are benign by definition, and after the advent of percutaneous ethanol injection (PEI), in most cases they do not require surgical treatment. Indeed, it is more than 20 years that PEI is successfully used as a safe, cheap, and effective treatment to significantly and permanently reduce the cystic volume, with a consequent relief for the patient [1–3]. Whereas data about efficacy of PEI are consistent, data about health-related quality of life (HRQL) are virtually absent.

In this report, we retrospectively evaluated the outcome, the pain perception, and the HRQL of a hundred of patients suffering from pure thyroid cysts who were submitted to PEI at our Institution.

## 2. Methods

We reviewed the data of patients treated in our Institution from 2012 to 2016. Criteria of inclusion were euthyroid patients who underwent PEI for a simple cystic lesion and having complaints for aesthetic or compressive troubles, negative cytologic result, and any cystic volume, but a number of PEI ≤ 3. Cystic volume was calculated using the ellipsoid formula (*D*1 × *D*2 × *D*3 × 0.52).

### 2.1. PEI Procedure

After disinfection of the skin and the use of anesthetic sprayed locally, using sonographic guidance, a 20-22-gauge needle is inserted into the cyst; after drainage of the almost total amount of cystic fluid, absolute ethanol is instilled to spread within the cyst. The injection is anyway stopped when ethanol leaks out of the cyst or if the patient complains of pain. 0.5 ml of 5% lidocaine is infused before needle extraction. The result of each PEI was controlled 1–4 weeks after, and PEI was repeated until we obtained a complete reabsorption to a reduction of at least 50% compared to baseline. Patients were requested to grade the sensation of pain suffered with the alcohol injection immediately after the procedure, using a visual scale of 10 cm between the absence of pain (0) and excruciating pain (10) in which each 1/3 of the scale corresponded, from the lowest to the highest, to the following categories: mild pain, moderate pain, and severe pain (the pain reported by the patient was the one in relation to the procedure and not the one experienced during the extraction of the needle). HRQL was assessed using the SF-12 Health Survey. The SF-12 Health Survey is a reduced form of the more extensive SF-36 questionnaire but is reliable as well, is more simple, and requires only a few minutes to complete it. The SF-12 uses 12 questions to cover 8 health domains: physical functioning, role limitations due to physical health, bodily pain, general health, vitality, social functioning, role limitations due to emotional problems, and mental health. The results can be summarized as a physical component summary (PCS-12) and a mental component summary (MCS-12). Higher scores indicate better health status.

### 2.2. Statistical Analysis

The comparison of parametric quantitative variables between groups was analyzed with Student's *t*-test and nonparametric variables with the Mann–Whitney* U* test. Qualitative variables were evaluated with the chi-square test. The comparison of volumes at different periods was conducted with the Friedman test, confirmed with nonparametric multiple comparison for dependent data. Multiple linear regression analysis was used to determine factors independently predictive of the percent nodule volume reduction. Factors entered into the model included age, sex, and basal nodule volume. Statistical Package for the Social Sciences (SPSS) version 16 was used for statistical analysis.

## 3. Results

**Figure 1:**
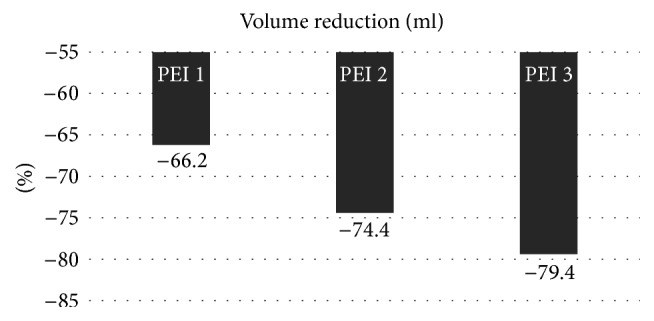
Volume reduction of thyroid cyst.

**Figure 2:**
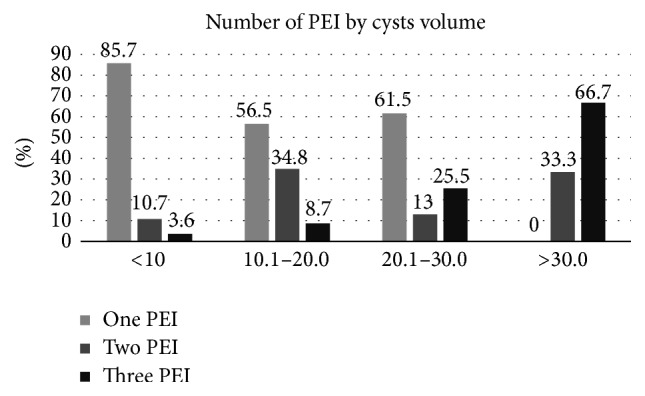
Number of percutaneous ethanol injections (PEI) practiced for thyroid cysts divided by baseline volume.

In the study period, 101 patients were collected (30 male and 71 female); mean age was 42.3 ± 12.9; mean cystic volume was 14.8 ± 15.5. Each patient underwent 1.4 ± 0.7 PEI. 56/101 (55.4%) of cysts had volume ≤ 10 ml, 23 (22.8%) 10.1–20.0 ml; 13 (12.9%) 20.1–30.0 ml, 9 (8.9%) > 30.0 ml (≤10 ml versus other volumes, *P* < 0.01). The baseline volume was significantly different among the four groups (*P* < 0.01). The number of PEI was significantly higher in the >30.0 ml group versus the other groups (*P* < 0.05). As expected, the higher the fluid drained was, the larger the cyst was (>30.0 ml group versus 20.1–30.0 ml group versus 10.1–20.0 ml group and versus ≤10 ml group; *P* for trend < 0.01), whereas the alcohol injected was significantly higher only at the first PEI in the >30 ml group (*P* < 0.05). The >30.0 ml group obtained the smallest percent reduction versus baseline after the first PEI (*P* < 0.05), whereas the ≤10.0 ml obtained the greatest percent reduction versus baseline after the third PEI (*P* < 0.05) (Table 1). In the whole group of patients, the volume reduction was 66% after the first, 74.4% after the second, and 79.4% after the third PEI treatment (*P* < 0.01 versus baseline) (Figure 1). A positive correlation between the volume and the number of PEI treatments was found: the vast majority (85.7%) of cysts ≤ 10 ml were cured by just one PEI, whereas 33.3% and 66.7% of cysts > 30 ml required 2 and 3 PEI, respectively (*P* < 0.01) (Figure 2). Multiple linear regression revealed that, among the considered parameters, only the baseline volume was predictive of response to PEI (*P* < 0.01).

The sensation of pain reported during PEI was virtually absent (78.3%), mild (17.4%), or moderate (4.1%). In all cases, this was a transient sensation of pain with relief within minutes. No patient felt intense pain and none suffered other complications. PCS-12 and MCS-12 both significantly improved from pre- to the posttreatment (50.1 ± 8.3 versus 54.7 ± 5.4 and 39.5 ± 13.1 versus 48.5 ± 6.8, respectively; *P* < 0.01).

## 4. Discussion

PEI is an outpatient procedure that has been used for different types of thyroid lesions like solid nodules, autonomously functioning nodules, and partially cystic nodules, but it has been demonstrated to be mainly successful in cystic lesions [4–6]. Data of the literature demonstrated that cystic volume can be significantly reduced and in some cases totally reabsorbed [7, 8]. Indeed, the updated guidelines released by the American Association of Clinical Endocrinologists/Associazione Medici Endocrinologi discourage PEI “for solid nodules, whether hyperfunctioning or not” and recommend it “as first-line treatment for cystic lesions.” The same guidelines define PEI as “a safe and effective outpatient therapy for thyroid cysts and complex nodules with a large fluid component” [9]. This retrospective study confirmed that PEI is effective and safe in attenuating or eliminating local discomfort or cosmetic concerns due to thyroid cysts but also revealed that patients experience an improved quality of life. This is the largest series of patients that demonstrates such a positive effect, beyond the structural changes induced by PEI. Similar results, although in a smaller number of patients, were obtained in a paper published by a Spanish group in 2015; in addition, Valcavi et al. demonstrated significantly improved HRQL also in patients, who suffered from solid nodules and underwent radiofrequency ablation [10, 11].

In this study, we selected simple cysts only, limiting the series of patients to those who underwent no more than 3 PEI. This is because we intended to select homogeneous group of thyroid lesions in order to evaluate the net effect induced by PEI in cyst shrinkage, without any “interference” due to any solid component of the nodule. Data clearly demonstrated that PEI is particularly (but not only) effective in cyst ≤ 10 ml, where a single PEI treatment is able to definitely eliminate the cyst. In more than half of cases, a single PEI is also effective for cysts of 10–30 ml, whereas, for those >30 ml, three PEI are necessary in 2/3 of cases. From these few figures, one would argue that considering PEI, as soon as cosmetic concerns or local discomfort appears, is a reasonable choice. PEI is also confirmed to be a safe procedure that is usually practiced in an office setting and does not require high-tech or expensive equipment; in skilled hands, it does not entail specific dangers, and the occasional pain felt by the patient due to ethanol leakage into subcutaneous tissues is usually mild and transient [12]. In relation to PEI side effects, some authors suggest that ethanol injection should be followed by aspiration of ethanol-mixed fluid, but no difference in adverse events nor in cystic volume reduction was observed between injecting and leaving a small amount of ethanol inside the cyst versus aspirating ethanol-mixed fluid; our clinical practice confirms that no serious adverse events occur when aspiration of ethanol-mixed fluid is not practiced [13]. Differently from most of studies, we used to inject small amounts of ethanol; indeed, no study demonstrated that the rate of success in cysts shrinkage correlates with the amount of injected ethanol; moreover, the injection of more elevated amount of ethanol may hesitate in more frequent and more intense pain experienced by the patient.

A limitation of the study is that the follow-up time was quite short (≤4 weeks). A longer follow-up could have shown an even better result than that observed in cystic shrinkage. Several studies demonstrated, although in solid nodules, that when the follow-up time is long (e.g., more than 12 months), the effect in terms of shrinkage is increased, probably due to a continuous sclerosing effect induced by ethanol over the time [14, 15].

In conclusion, PEI is effective for pure cystic thyroid lesions. In addition to that, PEI represents a safe treatment, with minimal, transient, and self-limited adverse events. The larger the cyst is, the greater the number of PEI needed to obtain a definitive result is. A significant improvement of HRQL in such treated patients provides a further support for this procedure.

## Figures and Tables

**Table 1:** Characteristics of thyroid cyst and percutaneous ethanol injection (PEI) procedures.

Volume (ml)	<10.0	10.1–20.0	20.1–30.0	>30	*P*
Baseline volume (ml)	6.4 ± 2.2	14.0 ± 2.5	26.0 ± 2.8	52.9 ± 24.9	<0.01

Number of PEI	1.2 ± 0.5	1.5 ± 0.7	1.5 ± 0.8	2.7 ± 0.5	<0.05

Fluid drained/session (ml)	PEI 1: 4.6 ± 2.1 PEI 2: 2.4 ± 1.6 PEI 3: 2.1 ± 0.1	PEI 1: 9.8 ± 3.9 PEI 2: 6.2 ± 2.5 PEI 3: 2.1 ± 0.9	PEI 1: 16.8 ± 5.8 PEI 2: 9.0 ± 2.2 PEI 3: 4.0 ± 4.2	PEI 1: 42.9 ± 20.2 PEI 2: 20.7 ± 11.6 PEI 3: 12.9 ± 10.7	<0.01 <0.01 <0.01

Alcohol injected/session	PEI 1: 1.6 ± 0.7 PEI 2: 1.2 ± 0.5 PEI 3: 1.0 ± 0.1	PEI 1: 2.8 ± 1.0 PEI 2: 2.2 ± 1.0 PEI 3: 1.0 ± 0.1	PEI 1: 3.4 ± 1.0 PEI 2: 2.2 ± 1.0 PEI 3: 2.0 ± 1.4	PEI 1: 6.0 ± 4.1 PEI 2: 3.1 ± 1.5 PEI 3: 2.4 ± 1.5	<0.05 NS NS

Volume reduction/session (%)	PEI 1: 67.6 PEI 2: 77 PEI 3: 93.1	PEI 1: 65.2 PEI 2: 68.6 PEI 3: 76.2	PEI 1: 72.1 PEI 2: 74.4 PEI 3: 79.2	PEI 1: 51.4 PEI 2: 78.2 PEI 3: 75	<0.05 NS <0.05

## References

[1] Verde G., Papini E., Pacella C. M. (1994). Ultrasound guided percutaneous ethanol injection in the treatment of cystic thyroid nodules. *Clinical Endocrinology*.

[2] Zingrillo M., Torlontano M., Chiarella R. (1999). Percutaneous ethanol injection may be a definitive treatment for symptomatic thyroid cystic nodules not treatable by surgery: Five-year follow-up study. *Thyroid*.

[3] Bennedbæk F. N., Hegedüs L. (2003). Treatment of recurrent thyroid cysts with ethanol: a randomized double-blind controlled trial. *Journal of Clinical Endocrinology and Metabolism*.

[4] Lippi F., Ferrari C., Manetti L. (1996). Treatment of solitary autonomous thyroid nodules by percutaneous ethanol injection: Results of an Italian multicenter study. *Journal of Clinical Endocrinology and Metabolism*.

[5] Zingrillo M., Collura D., Ghiggi M. R., Nirchio V., Trischitta V. (1998). Treatment of large cold benign thyroid nodules not eligible for surgery with percutaneous ethanol injection. *Journal of Clinical Endocrinology and Metabolism*.

[6] Bennedbæk F. N., Nielsen L. K., Hegedüs L. (1998). Effect of Percutaneous Ethanol Injection Therapy. *The Journal of Clinical Endocrinology & Metabolism*.

[7] Valcavi R., Frasoldati A. (2004). Ultrasound-guided percutaneous ethanol injection therapy in thyroid cystic nodules. *Endocrine Practice*.

[8] Del Prete S., Caraglia M., Russo D. (2002). Percutaneous ethanol injection efficacy in the treatment of large symptomatic thyroid cystic nodules: Ten-year follow-up of a large series. *Thyroid*.

[9] Gharib H., Papini E., Paschke R. (2016). AACE/ACE/AME task force on thyroid nodules. american association of clinical endocrinologists, associazione medici endocrinologi, and european thyroid association medical guidelines for clinical practice for the diagnosis and management of thyroid nodules: executive summary of recommendations. *Journal of Endocrinological Investigation*.

[10] Reverter J. L., Alonso N., Avila M., Lucas A., Mauricio D., Puig-Domingo M. (2015). Evaluation of efficacy, safety, pain perception and health-related quality of life of percutaneous ethanol injection as first-line treatment in symptomatic thyroid cysts. *BMC Endocrine Disorders*.

[11] Valcavi R., Tsamatropoulos P. (2015). Health-related quality of life after percutaneous radiofrequency ablation of cold, solid, benign thyroid nodules: a 2-year follow-up study in 40 patients. *Endocrine Practice*.

[12] Raggiunti B., Fiore G., Mongia A., Balducci G., Ballone E., Capone F. (2009). A 7-year follow-up of patients with thyroid cysts and pseudocysts treated with percutaneous ethanol injection: volume change and cost analysis. *Journal of Ultrasound*.

[13] Kim D. W., Rho M. H., Kim H. J., Kwon J. S., Sung Lee S. W. (2005). Percutaneous benign cystic is aspiration of fluid advantageous?. *American Journal of Neuroradiology*.

[14] Kim J. H., Lee H. K., Lee J. H., Ahn I. M., Choi C. G. (2003). Efficacy of sonographically guided percutaneous ethanol injection for treatment of thyroid cysts versus solid thyroid nodules. *American Journal of Roentgenology*.

[15] Lee S. J., Ahn I.-M. (2005). Effectiveness of percutaneous ethanol injection therapy in benign nodular and cystic thyroid diseases: Long-term follow-up experience. *Endocrine Journal*.

